# On Cider and the Vegetable Acids as Preventive of Cholera

**Published:** 1856-04

**Authors:** J. H. Tucker

**Affiliations:** Hon. Secretary to the Epidemiological Society.


### ON THE PROPHYLAXIS OF CHOLERA BY SOME OF THE VEGETABLE AND MINERAL ACIDS.

By J. H. TUCKER, Esq., Hon. Secretary to the Epidemiological Society.

In a paper I had the honour to read before the Epidemiological Society in July 1854, "On the use of Vegetable and Mineral Acids in the Treatment, Prophylactic and Remedial, of Cholera and other Epidemic Disorders of the Bowels," the mineral acids were dwelt upon in relation to diarrhœa, choleraic diarrhœa, cholera, and dysentery, as were also some of the vegetable acids.

I propose on this occasion to speak of the vegetable acids, as preventive of diarrhœa, choleraic diarrhœa, and cholera; and chiefly on the products of the apple—cider, and cider vinegar, when the first and second stages of fermentation have been completed.

Mr. Hunt first drew my attention in 1849 to the alleged exemption of some of the cider districts in Herefordshire from cholera, and this induced me to make inquiries of some medical men in and near my native village in Somersetshire, in order to ascertain from them whether the like exemption existed there, cider being the common beverage of the inhabitants. Replies were received from two gentlemen, which were published in the *Lancet* for July 30th, 1850; but it was not until I had read Mr. Herapath's observations, published in the *Lancet* for August 1851, on the exemption of cider districts from cholera, that I began to think seriously of cider as a prophylactic of cholera.

Since the reading of my first paper, I have endeavoured to obtain further information on the subject, and trust the result of my exertions will afford some degree of satisfaction.

I may first be permitted to allude to certain facts and observations bearing on the subject which have already been published by accredited authors. Dr. Aiken, in his work on cholera, published in August 1854, mentions the acids in relation to that disease. After having dwelt on the prophylactic powers of quinine and wine in fever, and having alluded to Dr. Bryson's work on the subject, he thus continues:—"It is quite possible that certain remedies of this class may induce such changes in the blood, as shall be either incompatible with, or at least strongly resist the primary changes induced by the morbific influence of the cholerac poison over the fluid. It has been supposed by some that certain acids exercise this power; and, although the data adduced are perhaps too scanty to warrant any definite conclusion, yet, from the hints already furnished, there is reason to expect a satisfactory result."

Dr. Headland, at page 129 of the second edition of his work "On the Action of Medicines on the System," states:—"It appears likely that the catalytic action of the vegetable acids consists in a certain illunderstood control over the progress of various cachexies and blood degenerations."

Among others it has been asserted, apparently upon reasonable amount of evidence, that these acids afford a sort of exemption from liability to Asiatic cholera. Again, at page 222, Dr. Headland says—"It is possible that Asiatic cholera may be connected with a rapid and fatal degeneration of the blood, produced by some septic influence; and there seems to be much reason for supposing that those who are accustomed to the use of the vegetable acids as articles of diet, as in the cider districts of England, are rendered thereby less liable to the attack of this disease."

Mr. Hingeston, of Brighton, has alluded to one of the vegetable acids as prophylactic of cholera. In the *Association Journal* for June 29th, 1853, will be found a letter from that gentleman on the probable recurrence of cholera this summer. Mr. Hingeston states,—"Indigestion, with heat at the præcordium, is again complained of; it appears to me that there is a want of acid in the blood, and that this form of dyspepsia is relievable by the vegetable acids, such as lemon juice."

In the pursuit of my object, I have had occasion to search for the opinion of authors who have written on the plague, a disease which Dr. Southwood Smith (in his Board of Health Reports) maintains to be, with yellow fever, allied to cholera. I find it stated, that the adoption of certain measures saved the lives of some who were, years gone by, subject to the influence of plague; why therefore need we despair that similar ones may be found to protect others from the cholera? Lemon-juice, vinegar, spirit or oil of vitriol, were among those which were adopted by good authorities, both as preventive and remedial of the plague.

It has been remarked that when diarrhœa ensued in patients attacked by plague, death was almost certain; whereas constipation was a favourable sign.

The value of sulphuric acid in preventing diarrhca and choleraic diarrhœa from passing into confirmed cholera, has been already tested. The *Lancet*, for October 3rd 1855 remarks thus ("On the Supplemental Report of the Medical Council of the General Board of Health." "On the Results of the Different Methods of Treatment of Epidemic Cholera throughout England and Ireland in 1854): — The most interesting and important portion of the series to the medical practitioner is the last, which gives the results of the different methods of treatment pursued in epidemic cholera."

In 1542 cases, the order of failure of remedies to prevent the promonitory diarrhœa from passing into confirmed cholera, would appear to have been catechu, kino, etc., with the employment of which the failures were fifty per cent, followed in order by salines, eliminants (castor-oil), calomel, calomel and opium, stimulants and astringents; and in this respect the treatment with sulphuric acid appears to have answered its purpose most fully, the percentage of failure with sulphuric acid and opium being set down at 5 per cent.

The value of vinegar and lemon-juice as preventives of cholera has yet to be shown. The fruit of the lemon-tree is too well known to need any eulogy from me. Its renown as an antiscorbutic none can (I believe) dispute, but whether it will prove hereafter to be protective from cholera experience must teach. The value of the juice of the lemon and of other fruits, save the apple, as regards diarrhœa and cholera, I shall leave to others to investigate.

I will now proceed to read some letters and other documents from medical gentlemen now living chiefly in Devonshire, Somersetshire, and Herefordshire, the three principal cider counties in England, in reference to the subject on which I am specially treating. Two of the letters were read by me on a former occasion, and I find I cannot well avoid reading them again on the present one. The first to be read was written by Mr. Sharpe, of Wedmore, Somersetshire, who had resided in that village but for a short period when he forwarded the communication to me in 1849. Mr. Sharpe succeeded Mr. Hancock in practice, whose communications will also be read with respect to cholera in 1832. Mr. Hancock at that period was parish surgeon. Mr. Hancock left England for America on family matters, he has lately returned to Wedmore.

Mr. Sharpe's letter, dated Dec. 1849, is as follows:—

"I beg to state, in answer to your inquiries respecting the late visitation of cholera, that I believe, in the parish of Wedmore, comprising a population of about 4,000, there have not been more than three cases of true Asiatic cholera, and very few cases of diarrhœa. Why such was the case I cannot give an opinion; for, in some of the districts, the inhabitants are both badly fed and clothed. The principal beverage is acid cider, of which they drink an immoderate quantity. The habitations of the poor are of the most wretched description. I am led to believe, from what little I can glean, there were only a few cases of the disease in 1832."

Mr. Millard, of Churchill, Somersetshire, wrote thus in January 1850:—

"I have been surgeon for eight years to district No. 5 in the Axbridge union, consisting of the parishes of Churchill and Windscombe; and during that period there has not been a single case of cholera. Cider is chiefly drank; which, in my opinion, is a more wholesome beverage than malt liquor. The air in this locality is particularly healthy, and the dwellings of the poor not overcrowded. The population is about 3,000."

My observations on these communications, read in my former paper, and published in the *Lancet*, July 1850, and in 1854, are as follows. Wedmore lies in a valley, on a level with the Cheddar Moors, where, in days gone by, ague and typhus were frequent; there, according to Mr. Sharpe's account, three cases of true Asiatic cholera came under observation, and a few cases of diarrhœa. The position of the poor, their habitations, and the locality, were such as to encourage the spread of the disease; yet only three cases were known, in a population of 4,000. Can the merit here be awarded to the cider?

Churchill is located on high ground, the air is salubrious, and the habitations of the poor not overcrowded; it is distant eight miles from Wedmore, and about fourteen from Bristol and Bedminster, where cholera raged to a fearful extent. To what cause is Churchill indebted for its exemption? Has cider, as a prophylactic, any claim?

The following is extracted from a note addressed to Dr. Hastings (now Sir Charles Hastings) by Dr. Lingen, of Hereford, dated November 9th, 1849.

"The overabundance of vegetation in this county, the free use of cider, and the almost contemptuous disregard of ventilation and sewerage evinced in these remote districts, are circumstances, one would say, *à priori*, highly calculated to imbibe and retain the scourge. We desire to thank God for our freedom from it; nevertheless, we would gladly discover any conducing means by which our immunity has been effected. You know better than I do the nature and quality of our surface as a country. We are on the old red sandstone, and have lime here and there; we have a clayey soil, interspersed with gravel. It may be that, so far from cider being productive of such a disease, it tends to keep the system in good order. I am highly impressed that it is really a wholesome drink, and less adulterated than beer."

As I find this to be the only communication from Herefordshire, among others placed in my hands by Mr. Hunt, I will direct attention to page 51 of the Registrar-General's Report on Cholera in 1848 and 1849, as follows: "In the county of Hereford, only *one* death from cholera was registered in 1849. A labourer's son, aged 9, died of cholera at Bargates, Leominster, on September 30th." I have since ascertained that the son of the labourer contracted the disease in another county, and was removed in a dying state.

"This county lies high; the population is scattered over the country, and engaged in agriculture; it is out of the line of railways. The common drink of the people is cider."

The following is copied from a note addressed to Mr. Hunt, by R. L. Pennell, M.D., Exeter, dated Jan. 29th, 1850.

"In reply to your first question, whether cider is the usual drink of the labouring population in Exeter, I have to say that beer is the common drink of the soldiers of the 82nd Regiment, whilst in the barracks at Plymouth, and also since their arrival in the barracks near this city. I have reason to believe that the large proportion of persons attacked with cholera in this city were beer-drinkers."

Mr. Hunt also placed in my hands some voluminous answers to questions with respect to cholera, asked in a printed form, by the Provincial Medical and Surgical Association. Among them, I find, "Observations on the late visitation of cholera in the city of Exeter", by Dr. Pennell, which terminate thus: "I cannot recommend any method of treatment in this disease from my own experience; but, as the subject of cider has been brought forward, I will briefly state a communication made by Mr. Tucker, a practitioner of great experience in his profession, and of long standing in this city (Exeter). Mr. Tucker says, that in 1832, being concerned in the treatment of the poor, a very large number of cholera patients came under his notice. The usual and varied remedies were employed with so little success, that he became dissatisfied with them all; so that he resolved to attend to the wishes of his patients, and let them have whatever they asked for. He mentions a young woman, aged 18, to whom he was summoned in August 1832, at seven in the evening. She had been taken ill about an hour, with all the characteristic symptoms of Asiatic cholera, such as vomiting, and purging of rice-coloured fluid. When he saw her, the powers of life seemed all but gone, extremities cold, no pulse, weak action about the heart, and the skin perfectly blue. Brandy and ammonia were first given, which she rejected; and warmth was applied to various parts of the body. At this time she asked eagerly for cider, in a low whisper, scarcely audible. Half a pint was given with some ginger; she drank it with avidity, and asked for more: another half pint was given. Mr. Tucker then left her, with directions that the warmth to the body should be continued, and that she might take as much cider as she called for. The next morning she was much better; the skin was warm, with a tendency to become moist, and the blueness was much less. From this time she gradually recovered, and is now living. The quantity of cider she took during the first night was seven pints.

"Within a few days, in the same neighbourhood, another case of cholera occurred. The same treatment was adopted; five quarts of cider with grated nutmeg were taken in twenty-four hours; all the symptoms yielded; and the patient (a male) recovered, and went about in the open air. A fortnight after, he had another attack of cholera, of which he died. On this occasion, Mr. Tucker did not attend him; but he ascertained, upon inquiry, that no cider was given to him.

"Such is the account of two cases successfully treated by this liquor. Of course no conclusion can be drawn from them, further than that the remedy is well deserving further trial, particularly as the prophylactic and therapeutic qualities of this beverage are become the subject of medical inquiry.

"It has been thought by some, that the quantity of rough cider consumed by the peasantry of this county is the cause of rheumatism prevailing so much among them, in fact, a large proportion of the medical cases received into our hospitals consist of this class of persons afflicted with the chronic form of the disease. I do not, however, think that this opinion is shown to be well founded. The constant exposure to wet and cold is quite sufficient to account for its prevalence among them."

When I commenced this paper, I was not aware that the value of cider was known as a remedy for cholera as far back as 1832. Mr. Sharpe, in his communication to me in 1849 remarked: "I am led to believe, from what little I can glean, there were only a few cases of the disease in this locality in 1832." Mr. John Hancock, of whom I have previously spoken in connexion with Mr. Sharpe, favoured me in May 1855 with the following relation:—"Practising in this neighbourhood in 1832, when Asiatic cholera first made its appearance in England, I was called to the majority of cholera cases at Wedmore, and I do not remember a single instance of this fatal epidemic having occurred in the better classes of society, otherwise those who were in the habit, and had the means of procuring the beverage common to this part of the country—cider. I attended fifty-three cases of severe diarrhœa and cholera, all among the poorer classes, and those females who had not the means, directly or indirectly, of procuring cider. How far cider acts as a prophylactic against the pest I am at this moment unprepared to speak; but should it again unfortunately be my lot to attempt to investigate the ravages of this fatal epidemic, I will not lose sight of your views, and will record any and everything bearing upon the point."

Mr. John Jackson Goodridge, surgeon, of Paignton, Devonshire, favoured me with the following in April 1845:—"I perfectly recollect writing the letter you mention, and am glad to have an opportunity of communicating to you my opinion and experience with regard to cider as a common beverage. Having practised in this part for the last forty years, I have had a great opportunity of observing the effect it has had on the labouring classes in this neighbourhood. Our parish has always been considered one of the healthiest in this county, being mild and free from dampness, and our soil being sandy we have not that great evaporation which is attributed to most parts of Devonshire. I think we number about two thousand seven hundred inhabitants, and I should calculate that from seven to eight hundred are labourers, whose daily drink is cider, each man taking his two quarts a day, and a more healthy set of men, it is impossible to see. They are most of them in friendly societies, and come under my immediate care. We have some few mechanics, who for the most part drink malt liquor, and I always find them much more intractable patients than those who drink cider.

"With regard to the prophylactic properties of cider there can be no doubt, as the neighbourhood was twice visited by cholera, and we were surrounded by the disease, and each time escaped, not having had a symptom of it. I think it would be not quite fair to place it all to cider; I, in some measure, attribute the exemption to our soil being very absorbent, dry, and sandy.

"The diseases most prevalent here are sciatica and rheumatism, which I think are caused in this way. Most of the labourers leave home of a morning with their dinners and a keg of cider, and take their meals by the side of a hedge, let the weather be what it may, and remain there an hour. I have often had occasion to observe that I have been called in late of an evening after a rainy day to attend upon those attacked.

"It has been remarked that a great number of our old labourers are lame, no doubt from the above cause. Generally speaking, they live to a good old age; and I have known men live to the age of ninety, the latter part of whose lives was daily spent in intoxication from cider. Not so with malt liquor. I generally find men who have lived freely on that beverage become bloated and die prematurely."

The next communication is from William Gillard, Esq., surgeon, of Totnes, Devonshire, March 20th, 1855.

"I have never known cider," says this gentleman, "the cause of any disease such as colica pictonum, or rheumatism, and certainly I may say I never knew it to produce cholera; and I believe I can confidently assert that the cider districts have been generally most free from that disease. I can also assure you that in many cases of dyspepsia I order an abstinence from malt liquor, at the same time recommending cider."

In my former paper, I quoted Dr. Mitchell of Dulverton, who had communicated to the *Lancet* on the subject of cider; and, being anxious to obtain further information, I wrote to that gentleman, and received from him a long article, a portion of which I will read, on the use of the vegetable and mineral acids, as employed by him.

"In answer to yours of the 24th instant, which did not reach me until this day, I have to inform you that I have used acids in diarrhœa, dysentery, etc., for these last forty years." Dr. Mitchell then alludes to his having used sulphuric acid in 1832, at the Redcross Street temporary cholera hospital in London, and states his reasons why and with what results, and thus continues. "You ask my opinion of cider in diarrhœa and dysentery. My treatment has always succeeded in dysentery, even when the patient has been abandoned under other prescriptions, and in such a state of prostration as to be unable to be taken out of bed; and with a frequent discharge of mucus, bloody, painful evacuations, and constant tenesmus, I have ordered and seen carried out as a dietary, cider *ad libitum* with only two small biscuits in twenty-four hours. This has been continued for weeks, until the disease has been completely removed. I have seen patients under this treatment regain strength, although kept on so limited a diet.

"About two years since, dysentery raged with great violence for a long time in the Cornwall Lunatic Asylum, destroying the lives of a number of its inmates. I was called in during its continuance. I ordered, as I always have most successfully in such cases, cider, excluding all other liquids, with only a very small quantity of toasted bread or biscuit, a line of treatment which had the desired effect in all cases, preventing any more deaths from diarrhœa or dysentery during my attendance, which has been frequently remarked upon since by Mr. Kendall, the member for East Cornwall, who is chairman of the visiting magistrates.

"The talented surgeon of this Asylum was appointed to Colney Hatch establishment; and in his report remarked how this generally very troublesome disease, in this, as well as in other houses of the sort, was kept under control by the treatment I have named—in fact, prevented—by substituting cider for ale as a drink. I have been told by patients that cider produces cramp. It always has a contrary effect, causing this painful state almost directly to cease.

"I called the attention of the Board of Health to this treatment more than once, which not being noticed except in an official way, caused me to direct the attention of the profession at large to its efficacy. Cholera has appeared at Bodmin in several instances, but in no case has it spread, only one death from it having taken place in the town; that of a woman taken out of a van in a dying state. I could name almost every town in cider districts in the west of England where cholera never could get a footing, except in places where ale is the chief beverage. I have no hesitation in saying that if there is a specific in diarrhœa (which I doubt), cider is that specific."

Mr. Gillard of Totnes, in August 1853, favoured me with a second communication, which I will read.

" I have made inquiries of many medical men in our neighbourhood, and none of them have ever known cider produce an attack of cholera; indeed, we all consider the cider districts most free from its visitation. In 1832, we had many cases in Totnes; about fifty reported, and sixteen deaths, mostly females; I should from recollection say, that not one of those attacked drank cider.

" Cholera has not appeared since that date in my district. Brixham has had two visitations since; Dartmouth, two; South Brent, one; as well as a small village near Ashburton. In neither place should I say there was ever much cider drank. Good fermented cider is undoubtedly wholesome; but in that you get from the manufacturer, or cider merchant, the process of fermentation is checked. Query, does that benefit it? As for cider from the press, or what you call *sweet cider*, when a boy, I perfectly recollect a very favourite amusement was sucking it through a reed, and then we called it 'nimble go through.' "

In addition to these, I have received communications from Mr. Kellock of Totnes; Mr. Woollen of Wedmore, Somerset; Dr. Turner of Kensington; and Mr. Davis of Pershore, and others; but as these gentlemen have written on the general rather than on the special properties of this beverage, I reserve their valued assistance for a future occasion.

In July last, I forwarded to the *Lancet*, and *Medical Times*, the following question, which appeared in these journals for July 14th, 1855.

" IS CIDER A PROPHYLACTIC OF CHOLERA?

" Sir,—Having reason to believe that good fermented cider is a prophylactic of cholera, I should feel obliged if any one of your readers who may have attended a case of cholera, in a cider drinking patient, will kindly inform me of the circumstance, either through the medium of your journal or by private communication. No such case has come to my knowledge."

I am not aware whether any communication on the subject has appeared in either of the journals since the question was asked. The following is the only one sent to me.

From E. Snell, Esq., surgeon :—

"18, Crown Row, Mile End Road, July 16, 1855.

"My eyes have just fallen on a paragraph in last week's *Medical Times and Gazette,* requesting information regarding cholera affecting cider drinkers. In this respect, I am sure you will find a difficulty.

"I received my early professional education in Devonshire, and was afterwards in practice in the same county for some years, and more particularly in a locality where the purest cider is drank to a very great extent both by masters and labourers. I never saw or heard of a case of cholera affecting either, very seldom even a case of diarrhœa, excepting in those persons having taken cider before it was in a fit state to drink."

In the *Medical Times and Gazette* for Oct. 27th, 1855, will be found the following letter from me.

"IS CIDER A PROPHYLACTIC OF CHOLERA?

"Sir,—I beg to offer you my sincere thanks for your having given insertion in your journal of July 14th to my query on the above subject. I have nowhere noticed that the question has been negatived; and with your permission I will now entreat your readers to favour me, either through the medium of your journal or by private letter, with any facts they may possess with respect to the value of good cider, in a medicinal, dietetic, or prophylactic (of cholera) point of view, or with facts adverse to its use; my object being to lay before the profession as early as possible all the information I can glean on the subject.

"At the present moment, I have every reason to hope that I shall have it in my power to remove the prejudices which have so long existed against this wholesome health-producing, and oftentimes delicious, beverage."

Since the publication of the foregoing, I have received no information for or against the use of cider.

A communication has, however, reached me from Mr. Hancock of Wedmore, on cider, which I will read after I have made a few observations on Dr. Varrentrapp's communication on cholera, read before the Society in August last.

In that communication, Dr. Varrentrapp alluded to cider as having been drank by persons who afterwards died of cholera. The liquor Dr. Varrentrapp spoke of was styled by him "sweet cider" (before fermentation), and he concludes his observations thus:—

"In several cases that happened during the next months, errors in diet seem to have been the cause. In several instances, the disease was ascribed to the use of sweet cider, in larger or smaller quantities. At all events, we should not suppose in our town, where a very large quantity of cider is drank (100,000 to 150,000 hundred weight of apples being used for cider in a year), that cider could be of any use in cholera, or even in choleraic times. Assuredly, of all drinks, cider would be the first to be forbidden with us, as melons were in France in the times of epidemics of cholera."

I presume that the liquid which Dr. Varrentrapp styled "*sweet cider*" (before fermentation) was in reality not cider. Cider is the fermented juice of apples, not the juice of apples before fermentation, nor during the process of fermentation —in fact, it is doubtless the fermented, not the fermenting, juice. The apple juice recently expressed, and for some time after, to my knowledge, acts as a drastic.

Sweetwort is not ale, and sweetwort acts as an aperient. The juice of the grape is not wine until fermentation has ceased, and Dr. Varrentrapp stated that sweet wine, before fermentation, occasioned cholera. Treacle, sugar, and water, when mixed together, are not vinegar, until they become sour by fermentation.

Dr. Varrentrapp compares the cider of his country to the juice of the melon; the cider of this country has been sometimes likened to vinegar and water. I know not what condition the sweet cider in Dr. Varrentrapp's locality may be; but in some parts of England cider which has been well and properly fermented retains a fair amount of sweetness, and is both palatable and wholesome. I have read and heard that in some parts of Germany cider of a very inferior quality is vended.

Dr. Varrentrapp resides at Frankfort-on-the-Maine.

Dr. Virchow, who resides at Bonn, has likewise forwarded a communication on cholera to the Epidemiological Society, in which cider is named. Dr. Weber, to whom in the first instance the communication was sent, states that Dr. Virchow resided during the epidemic of 1848-49 at Berlin; during that of 1853-54 at Würzburg. Dr. Virchow writes "that Würzburg has never been seriously visited by the cholera, although other districts of Bavaria had not remained exempt. During the last epidemic several cases of cholera were imported from Munich, but the disease did not spread. The immunity from the disease was not confined to the neighbourhood of Würzburg, but comprised the greater part of the Maine district, and extended along the banks of that river to Frankfort, with the exception of a few sporadic cases (imported)."

To question No. 10, series v, issued by our Society, Professor Virchow replies thus:—

"That vinegar and acid wines are much used in the district of Würzburg and Aschschaffenburg, and that cider is a common beverage on the lower course of the Maine."

As Professor Virchow has named cider in conjunction with vinegar and acid wines, I presume such cider must be the acid or hard cider. Besides, I have conversed with those who have drank the acid cider on the continent. A great quantity of such cider, I am informed, is used by some wine merchants who imitate foreign wines. The same takes place in some parts of England. Hence many persons who would not be tempted to drink cider, have enjoyed it somewhat disguised and under another name.

In proof of this, I beg to refer to *A Treatise on the Falsifications of Food, etc.*, by John Mitchell. At page 118 of his work, Mr. Mitchell remarks thus:—"I may mention that, among the substances convertible into wine by the addition of colouring and flavouring matter, is cider, many thousand pipes of which, in a state not drinkable as cider, are manufactured into fictitious port wine."

During a visit in Somersetshire, in August last, a gentleman, Mr. W., not of the medical profession, said, in relation to cholera, that he thought he could name some cases which would serve to prove that cider, as a preventive of cholera, was not always to be relied on. He then mentioned four persons who had died of cholera at Godney, and who were, he understood, during lifetime cider drinkers. Messrs. Sharpe and Hancock, therefore, inquired into the matter; and on the 28th October, 1855, the following communication reached me from Mr. Hancock.

"I have at length made inquiries relative to the cases of cholera reported as having occurred at Godney, by Mr. W.; and, strange to say, the facts bear you out in your doctrine to the very letter.

"There were five persons attacked within a circuit of three hundred yards. A father and son, who had been teetotallers for the last twenty years of their existence, and men who strictly carried out the doctrine of total abstinence. Both fell victims to the plague in a few hours.

"Another man of the same faith, also fatal. The fourth case was that of a confirmed drunkard, 'a cider swiller,' and while in intense agonies from spasm, he constantly called out for cider. At last it was given to him. This man recovered, and is now living.

"In the particular case of Mrs. W., brought forward also by Mr. W., who almost died in his presence, the facts prove to be, that she also was a water drinker, and had never been known in the remembrance of her husband to drink cider."

Having thus placed before you, Mr. President and Gentlemen, that which I have considered to be the most important communications from medical authority, on the subject of cider, I will make a few observations on the juices of apples in the second stage of fermentation—cider vinegar. In my former paper, I suggested vinegar as a substitute for cider, as a prophylactic of cholera, supposing then that London and other populous places could not be supplied with a sufficient quantity of pure cider for consumption.

Various authorities were quoted by me as to the medicinal and dietetic properties of vinegar, and I dwelt somewhat on adulterations of that article. About that period, my attention was accidentally drawn to cider vinegar; and remembering that such was used by my forefathers, and by myself in early life, I resolved to give it a trial in my own family. For the last eighteen months cider vinegar only has been used at my table. Many of my patients and friends have highly approved of it, some preferring it to malt, or even wine vinegar. Cider vinegar is chiefly used as a condiment, and for pickling in cider districts, and it is frequently given to the poor for such purposes. A portion of the cider vinegar in my possession has been partly analysed by an analytical chemist at one of the metropolitan hospitals, who has thus reported on it.

"I have examined the vinegar you left at the hospital. Its acidity is due almost entirely to acetic acid. There is a minute proportion of sulphuric acid, and also of some crystalline acid, probably malic. The flavour of the distilled vinegar is very agreeable; the acetic æther flavour is modified, probably by the presence of malic æther in addition. I consider it in every respect a very good vinegar."

As far as my own experience goes, from observations made by me when in Somersetshire, and from replies to my questions, I am prepared to state that I believe cider vinegar to be a very wholesome vegetable acid, and one that medical men may with confidence recommend to their patients, both in a medicinal and dietetic point of view.

