# Errata

**Published:** 1856-07

**Authors:** 


### ERRATA.

At page 11 in the *Transactions of the Epidemiological Society* for April 1856, line 33, for June 29, 1853, read 1855; at page 16, line 32, for 1845 read 1855; and at page 19, line 4, for 1853 read 1855.

